# A Systematic Literature Review on the Application of Machine-Learning Models in Behavioral Assessment of Autism Spectrum Disorder

**DOI:** 10.3390/jpm11040299

**Published:** 2021-04-14

**Authors:** Nadire Cavus, Abdulmalik A. Lawan, Zurki Ibrahim, Abdullahi Dahiru, Sadiya Tahir, Usama Ishaq Abdulrazak, Adamu Hussaini

**Affiliations:** 1 Department of Computer Information Systems, Near East University, Nicosia 99138, Cyprus; nadire.cavus@neu.edu.tr; 2 Computer Information Systems Research and Technology Centre, Near East University, Nicosia 99138, Cyprus; 3 Department of Computer Science, Kano University of Science and Technology, Wudil 713281, Nigeria; adamu.hussaini2510@gmail.com; 4 Department of Medical Genetics, Near East University, Nicosia 99138, Cyprus; zurkiibrahim@yahoo.com; 5 College of Nursing and Midwifery, School of Nursing, Kano 700233, Nigeria; abdullahidahiru84@gmail.com; 6 Department of Pediatrics, Murtala Muhammad Specialist Hospital, Kano 700251, Nigeria; taheersadiyah@gmail.com; 7 Department of Emergency Medicine, Peterborough City Hospital, North West Anglia NHS Foundation Trust, Peterborough PE3 9GZ, UK; usamia12@gmail.com; 8 Crestic Laboratory, Universite de Reims, 51100 Reims, France

**Keywords:** autism spectrum disorder, screening, diagnosis, artificial intelligence, machine learning

## Abstract

Autism spectrum disorder (ASD) is associated with significant social, communication, and behavioral challenges. The insufficient number of trained clinicians coupled with limited accessibility to quick and accurate diagnostic tools resulted in overlooking early symptoms of ASD in children around the world. Several studies have utilized behavioral data in developing and evaluating the performance of machine learning (ML) models toward quick and intelligent ASD assessment systems. However, despite the good evaluation metrics achieved by the ML models, there is not enough evidence on the readiness of the models for clinical use. Specifically, none of the existing studies reported the real-life application of the ML-based models. This might be related to numerous challenges associated with the data-centric techniques utilized and their misalignment with the conceptual basis upon which professionals diagnose ASD. The present work systematically reviewed recent articles on the application of ML in the behavioral assessment of ASD, and highlighted common challenges in the studies, and proposed vital considerations for real-life implementation of ML-based ASD screening and diagnostic systems. This review will serve as a guide for researchers, neuropsychiatrists, psychologists, and relevant stakeholders on the advances in ASD screening and diagnosis using ML.

## 1. Introduction

Autism spectrum disorder (ASD) is a lifelong neurodevelopmental disorder associated with communication impairment, restrictive and compulsive behavior. According to the fifth edition of the diagnostic and statistical manual of mental disorders (DSM-5), the primary indicators for diagnosing ASD are deficits in social communication and the manifestation of repetitive and restricted patterns of activities, behavior, or interests [1]. The rising prevalence of ASD necessitates the need for early and cost-effective diagnosis to set the path for efficient, and appropriate treatment [2,3]. Moreover, early diagnosis of ASD leads to improved outcomes in communication and social interaction and guides parents to the right interventions in school, home, and clinic [4,5,6]. However, apart from the cost-ineffectiveness of the current diagnostic instruments, studies have indicated the delay of the clinical processes of diagnosing ASD [7,8,9,10]. Addressing these challenges lead to several suggestions, including the so-called quick and accurate Machine Learning (ML)-enabled ASD assessment systems [11,12,13,14]. The promising results realized with ML algorithms across various research fields motivated these suggestions and made it a vital step toward quick and cost-effective assessment of ASD symptoms.

The gap in the existing literature is the absence of a definitive explanation on the sufficiency and readiness of the ML models toward real-life implementation. Recently, there is an increasing number of studies on the development of ML models for diagnosing ASD based on either genetic [15,16], brain imaging [17,18,19], physical biomarkers [20,21,22,23,24], or behavioral data. However, despite the high evaluation metrics reported in the ML-based behavioral studies, there is little evidence on the clinical use of the resulting ML models [11]. Generally, apart from improving the accuracy metrics of the ML models, previous studies focused on improving diagnostic speed by reducing the model parameters using various dimensionality reduction techniques. Worthy of note, both the ML algorithms and the dimensionality reduction techniques are data-centric; they are independent of the conceptual basis upon which professionals build and utilize ASD assessment instruments [25]. Thus, the clinical validity of the resulting ML models could be explained based on the alignment of the data-centric techniques with the conceptual basis of diagnosing ASD. Nonetheless, other factors that might limit the clinical validity and real-life implementation of the models include the reported discrepancies within the data repositories [26,27].

The present review explores the advances in the application of machine learning in the behavioral assessment of ASD. Accordingly, recent articles were systematically reviewed on the application of machine learning models toward quick and accurate assessment of ASD. Based on the reviewed literature, we sought the answer on whether the recent findings could sufficiently translate to real-life implementation of ML-based ASD screening and diagnostic models. Nonetheless, previous literature reviews assessed the performance of ML models in ASD screening and diagnosis based on the common evaluation metrics of sensitivity, specificity, and accuracy, among others [25,28]. However, none of the existing literature reviews systematically analyzed the subject area and provided enough evidence on the readiness and sufficiency of the models toward real-life implementation of the ML-based systems. For instance, Song et al. [28] reviewed 13 relevant studies that utilized varying data types and discussed the possibility of achieving effective classification of ASD based on the study findings. Similarly, Thabtah [25] identified some limitations within the commonly employed research methodologies and proposed intuitive stages toward appending the ML models into ASD screening apps. In this work, key challenges were highlighted alongside the commonly utilized assessment tools, datasets, and data intelligence techniques, and solutions were suggested toward valid implementations of real-life ML-based ASD screening and diagnostic systems.

## 2. Methodology

### 2.1. Search Strategy

The present review involved a systematic search, which is conducted in October 2020. To identify the most relevant studies, the authors ensured careful planning and allocation of tasks at every stage of the systematic literature review. The search strategy was tailored to the four most popular scientific databases of the study field, namely, Web of Science, PubMed, IEEEXplore, and Scopus. Furthermore, the search query utilized includes the following terms “Autism Spectrum Disorder” OR “Autistic Disorder” OR “Autism” AND “Screening” OR “Assessment” OR “Identification” OR “Test” OR “Detection” AND “Machine Learning” OR “Artificial Intelligence”. The search filters covered a period of ten years from 2011 to 2020 and were limited to journal articles published in the English language. Beyond the above-mentioned databases, relevant publications were accessed from other databases on the advances in ASD assessment.

### 2.2. Selection Criteria

The article selection process was based on the PRISMA statement [29]. Relevant studies have utilized PRISMA in providing critical appraisal on the advances in the assessment of autism and other neuropsychiatric disorders [19,24,28,30,31,32,33]. The determining factor in the inclusion criteria involves any published full-text journal article on the use of ML in ASD screening or diagnosis. At the initial screening stage, after duplicates removal, the authors assessed the records against the inclusion criteria to decide on worthy articles for the systematic literature review. The decisions for inclusion/exclusion on the records were recorded in a separate column within the combined excel sheet imported from the databases. Thus, for records whose titles and corresponding abstracts aligned with the preset inclusion criteria, full-text articles of the studies were retrieved for the subsequent screening stage. In the next PRISMA screening stage, all the authors reviewed the downloaded papers, independently, to ascertain their relevance with the search query used, as well as the set research question. The authors utilized the WhatsApp discussion group in resolving disagreements in the selection process.

**Figure 1:**
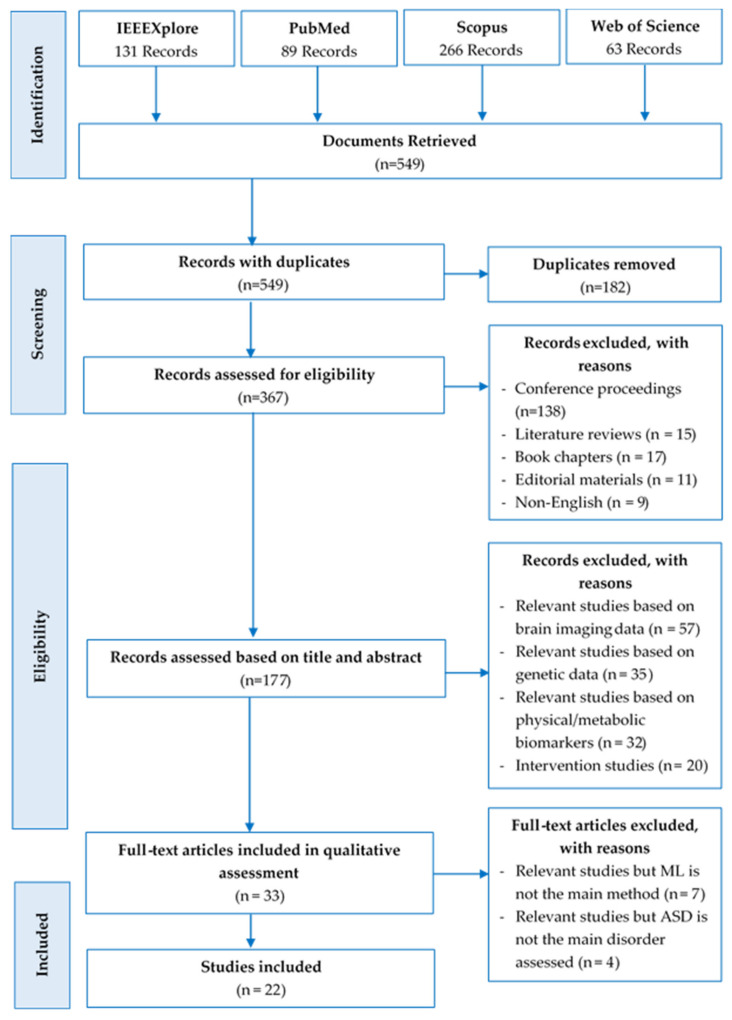
PRISMA flow diagram of the search results.

Specifically, three hundred and sixty-seven records were carefully assessed for eligibility. One hundred and eighty studies out of the 367 records were discarded, due to the following reasons: Book chapters (n = 17), conference papers (n = 138), editorial materials (n = 11), literature reviews (n = 15), not written in English (n = 9). The remaining one hundred and seventy-seven studies were further assessed; one hundred and forty-four records were eliminated because they are either based on brain imaging data (n = 57), genetic data (n = 35), or physical/metabolic biomarkers (n = 32), while others are intervention studies (n = 20). Consequently, thirty-three full-text articles were retrieved, read, and qualitatively assessed. Nonetheless, additional articles were excluded because ML is not the main method employed (n = 7), and ASD is not the main neuropsychiatric disorder assessed (n = 4). Finally, 22 studies met the inclusion criteria. The PRISMA flow diagram (Figure 1) summarized the above-mentioned systematic literature review process, and Table 1 itemized the key items of the inclusion and exclusion criteria of the study.

### 2.3. Quality Assessment

The authors carefully adhered to the planned, systematic literature review process to maintain the study’s quality. Particularly, at every phase of the systematic literature review, the authors ensured careful planning and allocation of tasks. The first author created an online Mendeley repository and monitored the progress of the review based on preset milestones to ensure that all tasks complied with the scheduled deadlines. The Mendeley repository was also used in keeping track of the data extraction stages, noting essential observations and sharing vital contents related to the study. The authors further upheld peer-reviewing at each phase of the study to enhance the systematic literature review. Nevertheless, unbiased and constructive assessments on the systematic approach used in this study were sought from external professionals on ASD diagnostic procedures with expertise in systematic literature reviews.

### 2.4. Data Extraction

As the final stage of the study’s PRISMA, the data extraction stage, 22 articles were appraised critically, and the following information was extracted from the studies:

- Author(s) (year),
- Number of citations,
- Source(s) of the research data,
- Data collection/assessment instrument,
- ML model(s)developed,
- Best performing model(s),
- The key finding(s).

## 3. Results

### 3.1. Descriptive Analysis on Trends and Status of the Study on ML in ASD Assessment

Based on the exported data, the trend of studies on the use of ML in the behavioral assessment of ASD showed the most cited references, the most cited journals, as well as citation and publication frequencies across the years.

**Figure 2:**
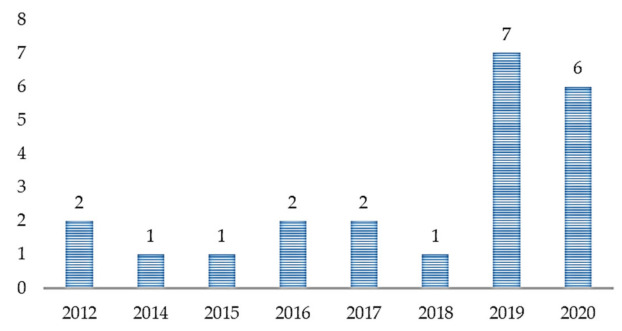
Article distribution over the years.

**Figure 3:**
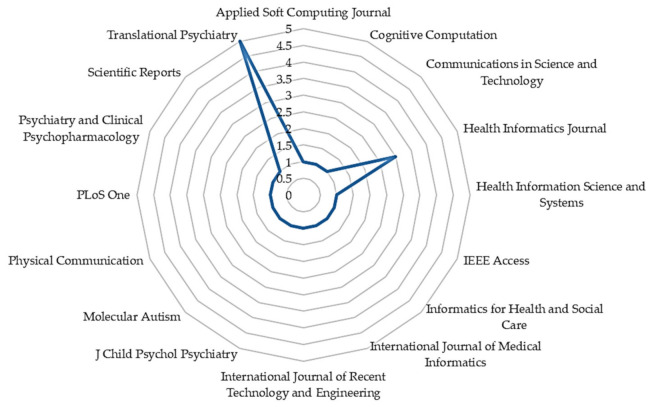
The number of articles published by journals.

With the increasing application of ML in healthcare studies, as shown in Figure 2, there are more publications on ML and ASD assessment. From 2012 to 2018, not so many studies cared about the application of ML in ASD assessment. However, with the recently increased patronage of ML techniques across various fields, there is an increasing demand for intelligent tools for accurate assessment of ASD. From Figure 3, most of the articles contributing to the area were published in Translational Psychiatry (n = 5), followed by the Health Informatics Journal (n = 3). The remaining fifteen journals depicted published one article, each.

**Figure 4:**
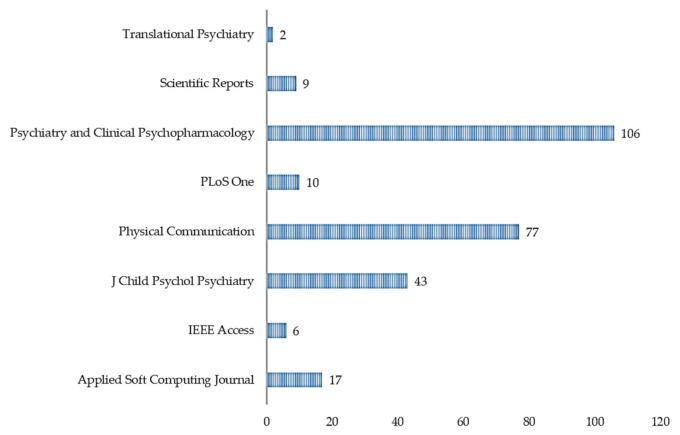
Sum of citations per journal.

**Figure 5:**
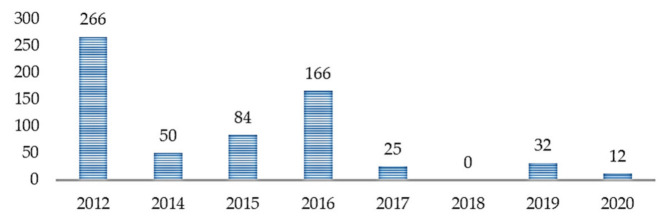
Number of citations across years.

Based on the citation data exported, as shown in Table 2, we can see that the most cited references are Wall et al. [34] (n = 160), Wall et al. [35] (n = 106), Duda et al. [36] (n = 89), Kosmicki et al. [37] (n = 84), and Bone et al. [38] (n = 77). Most of the significant references; with the highest number of citations, were published in Translational Psychiatry [34,36,37] (Figure 4, n = 408) in the years 2012 (Figure 5, n = 266), 2015 (Figure 5, n = 84), and 2016 (Figure 5, n = 166). Figure 4 highlighted the citation data of the eight most cited journals involved in the study; Translational Psychiatry (n = 408), PLoS One (n = 106), Journal of Children Psychological Psychiatry (n = 77), and so on.

### 3.2. Dimensionality Reduction Techniques

Most of the studies primarily aimed at streamlining the data collection instruments, followed by evaluating the performance of various ML algorithms on the streamlined datasets [35,37,39,40,41]. While various feature selection methods were applied in streamlining the most influential features of the data collection instruments from the datasets, other studies utilized various feature transformation techniques in reducing the input parameters. For instance, in the work of Puerto et al. [42], the inputs were fuzzified into membership values before applying the classification algorithms. Similarly, before implementing the classification models, Baadel et al. [43] and Akter et al. [44] transformed the inputs using clustering and feature transformation functions, respectively. Nonetheless, other studies employed a trial-error approach in selecting the most influential features. The trial-error approach involves repetitive evaluation of the ML models using a varying combination of the features; the most influential combination achieves superior results with fewer input parameters. Specifically, the studies utilized various feature selection techniques, including trial-error [13,34,35,39,45], Variable Analysis (Va) [46,47], information gain (IG) and chi-square testing (CHI) [48], sequential feature selection (SFS) [49], correlation-based feature selection (CFS) and minimum redundancy maximum relevance (mRMR) [12]. Additionally, ML-based feature selection techniques employed include recursive feature selection [40], sparsity/parsimony enforcing regularization techniques [50], stepwise backward feature selection [37], and forward feature selection [36].

### 3.3. Models Implementation

As shown in Table 2, the commonly implemented ML algorithms are Random Forest (RF) [12,43,47,51], Support Vector Machines (SVM) [37,38,40,49,50], Alternative Decision Tree (ADTree) [34,35,39,45], and Logistic Regression (LR) [13,37,48]. To achieve comparative results, most of the studies employed several algorithms, such as Adaboost, Artificial Neural Network (ANN), Linear Discriminant Analysis (LDA), Naïve Bayes, and K-Nearest Neighbor (KNN).

### 3.4. Data Collection/Assessment Instruments

The most utilized data collection instruments are AQ-10 [11,13,43,44,46,47,48,49,51,52], Q-CHAT-10 [11,44,46,52], ADOS [34,37,39,40,42,50], ADI-R [35,38,42], and Social Responsiveness Scale (SRS) [36,38,53]. Others include Autism Behavior Checklist, Aberrant Behavior Checklist, Clinical Global Impression [45], and MCHAT-based Pictorial Autism Assessment Schedule (PASS) [12]. Thus, the need for improving the reliability of these assessment instruments and ascertaining their relevance in ML modelling remains.

### 3.5. Sources of Data

The most prominent sources of data utilized in the studies include Boston Autism Consortium (AC), Autism Genetic Resource Exchange (AGRE), Simons Simplex Collection (SSC) [34,35,36,37,39,50,53], National Database for Autism Research (NDAR) [37,39], and Simons Variation In Individuals Project (SVIP) [37,39,50]. Other studies utilized data sets from ASDTest: Kaggle and UCI ML repository [11,13,43,44,46,47,48,49,51,52], Association of Parents and Friends for the Support and Defense of the rights of people with Autism (APADA) [42], PASS app [12], Ondokuz Mayis University Samsun [45], and ASD outpatient clinics in Germany [40]. To achieve standardized comparative results, there is a need for standardized ASD data repositories for machine learning studies [25].

### 3.6. Research Procedures

Apart from the common aim of streamlining the various data collection instruments followed by model evaluation, other studies focused on either optimizing the machine-learning algorithms [49,51], proposing input optimization techniques [43,44,46,47], or implementing ML-based screening apps [11,12]. For instance, Goel et al. [51] proposed Modified Grasshopper Optimization Algorithm (MGOA) for improved performance over common ML algorithms. The proposed MGOA (GOA with Random Forest classifier) outperformed other basic models and predicted ASD with approximate accuracy, specificity, and sensitivity of 100%. Similarly, Suresh et al. [49] proposed Differential Evaluation (DE) Algorithm to find the optimal solution of SVM parameters. The proposed DE tuned SVM achieved better performance over SVM, ANN and DE optimized ANN in classifying ASD. As stated earlier, apart from trial-error, studies employed either feature selection or transformation techniques for dimensionality reduction. For instance, Thabtah et al. [46] demonstrated the superiority of Va over IG, Correlation, CFS, and CHI in reducing AQ-10 items. Va derived fewer features, while maintaining competitive predictive accuracy, sensitivity, and specificity rates. A replicated study by Pratama et al. [47] produced a higher sensitivity of 87.89% in Adults AQ with RF and an increased specificity level of 86.33% in Adolescents AQ with SVM. Despite the good performance of the above-mentioned techniques in automating feature selection processes across various applications [54,55], none of the previous studies justified the conformity of the feature selection methods with the conceptual basis upon which professionals built and utilize ASD diagnostic instruments. Furthermore, unlike other medical diagnoses, the absence of definitive measures and medical tests for diagnosing ASD makes it difficult to numerical quantify the disorder based on few parameters. Notably, accurate assessment of ASD relied on the precise application of the commonly used behavioral scales built based on the knowledge and expertise of the professionals. Thus, applying human knowledge is imperative to reliable ASD diagnosis. Based on that, there is a need for quantifying the trade-offs of dimensionality reduction (ensuring fewer items for quick assessment) and validity (preservation of the human knowledge for correct diagnosis). Specifically, a machine-learning model built based on fewer behavioral features that do not sufficiently capture the human knowledge of the assessment instrument, will not be valid for clinical use. Thus, there is a need for applying dimensionality reduction techniques that professionals could track their ability to preserve the validity of the assessment instruments.

Nonetheless, various feature transformation techniques were equally utilized in the dimensionality reduction processes. For instance, Akter et al. [44] utilized three feature transformation techniques; Log, Z-score, and Sine functions, and evaluated the performance of nine different ML models on the transformed datasets. Log, Z-score, and Sine functions normalize data by converting excessively skewed entities into a normal distribution, converting features into −1 to 1 value range, and transforming instances to the Sine 0–2π value intervals, respectively. Akter et al. [44] recorded varying superior performances of the ML models, and the feature transformation approaches across the datasets. The feature transformations resulting in the best classifications were Z-score and Sine function on children, adolescents, and toddlers’ datasets, respectively. However, despite the reported improved performances of the ML models on the transformed datasets and the theoretical understanding of the capabilities of the transformation functions, studies have demonstrated how these transformations compromise the relevance of the original data to the transformed data [56,57,58,59]. Researchers ought to be mindful of the limitations in using these transformations in terms of the relevance of the original to the transformed data during results interpretation. For instance, Feng [59] demonstrated such irrelevancies between the statistical findings of standard tests performed on original and log-transformed data. Similarly, several studies have highlighted some of the pitfalls and inconsistencies in the application of Z-scores and its concepts that overlooked the meaning of the original data, its standard deviations, and confusing applications [56,57,58].

Recent studies further demonstrated how ML-enable ASD screening and diagnostic models could be developed, evaluated, and implemented. Recently, Baadel et al. [43] proposed Clustering-based Autistic Trait Classification (CATC), which identifies ASD-based traits’ similarity, unlike the commonly used scoring functions. CATC showed significant improvement in the ASD classification based on clustered inputs. Comparative evaluation of various classification algorithms showed better improvement with the Random Forest classifier. On the implementation of mobile apps for ASD screening, Wingfield et al. [12], and Shahamiri and Thabtah [11] embedded RF and CNN-based scoring models, respectively, while Thabtah [13] employed ML to validate ASDTest; a mobile screening app embedded with non-ML functions. In all the foregoing studies, the commonly used evaluation metrics are classification accuracy, sensitivity, and specificity. Specificity is the ratio of non-ASD cases that are correctly classified (i.e., true negatives rate) and sensitivity is the ratio of true ASD cases that are correctly classified (i.e., true positives rate), while classification accuracy is derived from sensitivity and specificity—as the measure of precisely classified cases from the total number of the cases.

**Table 2:** Information extracted from the articles.

Article/ Citations	Aim	Tool	Data Source	FS/FT	FS/FT Method	Modeling Algorithms	Key Findings
Goel et al. [51] C = 10	Proposed Optimization Algorithm for improved performance over common ML	AQ-10 (child, adolescent, adult)	ASDTest	-	-	GOA, BACO, LR, NB, KNN, RF-CART + ID3, * MGOA	The proposed MGOA (GOA with Random Forest classifier) predicted ASD cases with approximate accuracy, specificity, and sensitivity of 100%.
Shahamiri and Thabtah [11] C = 0	Implementation and evaluation of CNN-based ASD scoring system	Q-CHAT-10, AQ-10	ASDTest	-	-	C4.5, Bayes Net, RIDOR, * CNN	The performance evaluation showed the superior performance of CNN over other algorithms; indicating the robustness of the implemented system.
Thabtah and Peebles [52] C = 28	Demonstrate the superiority of Rules-based ML over other models	Q-CHAT-10, AQ-10 (child, Adolescent, adult)	ASDTest	-	-	RIPPER, RIDOR, Nnge, Bagging, CART, C4.5, and PRISM, * RML	Empirically evaluated rule induction, Bagging, Boosting, and decision trees algorithms on different ASD datasets. The superiority of the RML model was reported in not only classifying ASD but also offer rules that can be utilized in understanding the reasons behind the classification.
Wall et al. [35] C = 106	Streamlining ADR-I and evaluate ML performance	ADI-R	AGRE, SSC, AC	FS	Trial-error	* ADTree, BFTree, ConjunctiveRule, DecisionStump, FilteredClassifier, J48, J48graft, JRip, LADTree, Nnge, OneR, OrdinalClassClassifier, PART, Ridor, and SimpleCart	The best model utilized 7 of the 93 items contained in the ADI-R in classifying ASD with 99.9% accuracy.
Duda et al. [39] C = 50	Streamlining ADOS and demonstrate the superior performance of ADTree over common hand-crafted methods	ADOS	AC, AGRE, SSC, NDAR, SVIP	FS	Trial-error	ADTree	72% reduction in the items from ADOS-G with >97% accuracy.
Küpper et al. [40] C = 2	Streamlining ADOS and demonstrate the performance of SVM	ADOS	ASD outpatient clinics in Germany	FS	Recursive Feature Selection	SVM	SVM achieved good sensitivity and specificity with fewer ADOS items pointing to 5 behavioral features.
Wall et al. [34] C = 160	Streamlining ADOS and evaluate ML performance	ADOS	AC, AGRE, SSC	FS	Trial-error	* ADTree, BFTree, Decision Stump, Functional Tree, J48, J48graft, Jrip, LADTree, LMT, Nnge, OneR, PART, Random Tree, REPTree, Ridor, Simple Cart	The ADTree model utilized 8 of the 29 items in Module 1 of the ADOS and classified ASD with 100% accuracy.
Levy et al. [50] C = 21	Streamlining ADOS and evaluate ML performance	ADOS	AC, AGRE, SSC, SVIP	FS	Sparsity/parsimony enforcing regularization techniques	LR, Lasso, Ridge, Elastic net, Relaxed Lasso, Nearest shrunken centroids, LDA, * LR, * SVM, ADTree, RF, Gradient boosting, AdaBoost	With at most 10 features from ADOS′s Module 3 and Module 2, AUC of 0.95 and 0.93 was achieved, respectively.
Kosmicki et al. [37] C = 84	Streamlining ADOS and evaluate ML performance	ADOS	AC, AGRE, SSC, NDAR, SVIP	FS	Stepwise Backward Feature Selection	ADTree, * SVM, Logistic Model Tree, * LR, NB, NBTree, RF	The best performing models have utilized 9 of the 28 items from module 2, and 12 of the 28 items from module 3 in classifying ASD with 98.27% and 97.66% accuracy, respectively.
Thabtah [13] C = 31	Propose ASDTest; AQ-based mobile screening app, streamline AQ-10 items, and evaluate the performance of 2 ML models	AQ-10 (child, adolescent, adult)	ASDTest	FS	Trial-error	NB, * LR	Feature and predictive analyses demonstrate small groups of autistic traits improving the efficiency and accuracy of screening processes.
Thabtah et al. [46] C = 47	Demonstrate the superiority of Va over other FS methods based on the performance of ML models on the streamlined datasets	Q-CHAT-10, and AQ-10 (child, adolescent, adult)	ASDTest	FS	Va, IG, Correlation, CFS, and CHI	Repeated Incremental Pruning to Produce Error Reduction (RIPPER), C4.5 (Decision Tree)	Va derived fewer features from adults, adolescents, and child datasets with optimal model performance. Demonstrate the efficacy of Va over IG, Correlation, CFS, and CHI in reducing AQ-10 items
Thabtah et al. [48] C = 13	Streamlining AQ-10 and demonstrate the superior performance of LR over common hand-crafted methods	AQ-10 (adolescent, adult)	ASDTest	FS	IG, CHI	LR	LR showed acceptable performance in terms of sensitivity, specificity, and accuracy among others.
Suresh Kumar and Renugadevi [49] C = 0	Algorithm Optimization (improvement in accuracy compared to common ML)	AQ-10 (child, adolescent, adult)	ASDTest	FS	SFS	SVM, ANN, * DE SVM, DE ANN	DE optimized SVM outperformed ANN and DE optimized ANN in classifying ASD. DE is effective.
Pratama et al. [47] C = 0	Input Optimization using Va	AQ-10 (child, adolescent, adult)	ASDTest	FS	Va	SVM, * RF, ANN	RF succeeded in producing higher adult AQ sensitivity (87.89%), and a rise in the specificity level of AQ-Adolescents was better produced using SVM (86.33%).
Usta et al. [45] C = 9	ML Performance Evaluation	Autism Behavior Checklist, Aberrant Behavior Checklist, Clinical Global Impression	Ondokuz Mayis University Samsun	FS	Trial-error	NB, LR, * ADTree	The ML modeling revealed the significant influence of other demographic parameters in ASD classification.
Wingfield et al. [12] C = 3	Propose PASS; a culturally sensitive app embedded with ML model	PASS	VPASS app	FS	CFS, mRMR	* RF, NB, Adaboost, Multilayer Perceptron, J48, PART, SMO	PASS app overcomes the cultural variation in interpreting ASD symptoms, and the study demonstrated the possibility of removing feature redundancy.
Duda et al. [36] C = 89	ML Performance Evaluation in classifying ASD from ADHD	SRS	AC, AGRE, SSC	FS	Forward Feature Selection	ADTree, RF, SVM, LR, Categorical lasso, LDA	All the models could classify ASD from ADHD by utilizing 5 of the 65 items of SRS with high average accuracy (AUC = 0.965).
Duda et al. [53] C = 25	Improve models’ reliability using expanded datasets for classifying ASD from ADHD	SRS	AC, AGRE, SSC, and crowdsourced data	FS	-	SVM, LR, * LDA	LDA model achieved an AUC of 0.89 with 15 items.
Bone et al. [38] C = 77	Demonstrate the improved accuracy of SVM over common hand-crafted rules	ADI-R, SRS	Balanced Independent Dataset	FT	Tuned parameters across multiple levels of cross-validation	SVM	The SVM model utilized five of the fused ADI-R and SRS items and classified ASD sufficiently with below (above) 89.2% (86.7%) sensitivity and 59.0% (53.4%) specificity.
Puerto et al. [42] C = 17	Propose MFCM-ASD and evaluate its performance against other ML models	ADOS, ADI-R	APADA	FT	Inputs fuzzification	* MFCM-ASD, SVM, Random forest, NB	The superior performance of MFCM characterized by its robustness makes it an effective ASD diagnostic technique.
Akter et al. [44] C = 6	Compare FT methods and evaluate the performance of ML models on the transformed datasets	Q-CHAT-10, and AQ-10 (child, adolescent, adult)	ASDTest	FT	Log, Z-score, and Sine FT	Adaboost, FDA, C5.0, LDA, MDA, PDA, SVM, and CART	Varying superior performances of the ML models and FT approaches were achieved across the datasets.
Baadel et al. [43] C = 2	Input Optimization using a clustering approach	AQ-10 (child, adolescent, adult)	ASDTest	FT	CATC	OMCOKE, RIPPER, PART, * RF, RT, ANN	CATC showed significant improvement in screening ASD based on traits′ similarity as opposed to scoring functions. The improvement was more pronounced with RF classifier.

ASD, autism spectrum disorder; FS, feature selection; FT, feature transformation; ML, machine learning; ANN, artificial neural network; SVM, support vector machine; CNN, convolutional neural network; RF, random forest; LR, logistic regression; ADTree, alternative decision tree; LDA, linear discriminant analysis; MGOA, modified grasshopper optimization algorithm; BACO, binary ant colony optimization; NB, naïve Bayes; KNN, K-nearest neighbor; RIPPER, repeated incremental pruning to produce error reduction; ADOS, autism diagnostic observation schedule; ADI-R, autism diagnostic interview-revised; Q-CHAT, quantitative checklist for autism toddlers; AQ, autism quotient; SRS, social responsiveness scale; PASS, pictorial autism assessment schedule; AC, boston autism consortium; AGRE, autism genetic resource exchange; SSC, Simons Simplex Collection; NDAR, National Database for Autism Research; SVIP, Simons Variation In Individuals Project; APADA, Association of Parents and Friends for the Support and Defense of the Rights of People with Autism; MFCM, multilayer fuzzy cognitive maps; CATC, clustering-based autistic trait classification. * Best performing models.

## 4. Discussion

The search for cost-effective ASD assessment coupled with the global rise in ASD cases attracted the implementation of quick and accurate assessment measures based on data intelligence techniques, including machine-learning algorithms. Despite the various attempts in ML-based ASD assessment using functional magnetic resonance imaging (MRI), eye tracking, and genetic data, among others, the promising results based on behavioral data call for further research. For instance, Plitt et al. [60] found that ASD classification via behavioral measures consistently surpassed rs-fMRI classifiers. Accordingly, in line with the common research aim of the behavioral studies, various dimensionality reduction techniques were employed to improve the diagnostic speed of the resulting ML models. However, unlike the reduced dimensions, there is enough evidence on the good reliability, high internal consistency, and convergent validity between the common assessment instruments within large samples [61,62,63,64,65]. Furthermore, studies have ascertained the robustness of the common assessment instruments in the quantitative measurement of the various dimensions of communication, interpersonal behavior, and stereotypic/repetitive behavior associated with ASD. Therefore, it will be difficult to sufficiently measure the key dimensions of the instruments using the fewer items generated by the common dimensionality reduction techniques. For instance, while professionals interpret SRS scores based on the sum of its 65 items, Bone et al. [38], Duda et al. [36], and Duda et al. [53] implemented SRS-enabled machine-learning models with at most 5, 5, and 15 items, respectively. Specifically, Duda et al. [36] and Duda et al. [53] focused on classifying ASD from ADHD using the SRS data from AC, AGRE, SSC. Duda et al. [36] implemented ADTree, RF, SVM, LR, Categorical lasso, and LDA models and achieved the highest area under the curve (AUC) of 0.965 in classifying ASD from ADHD by utilizing five of the 65 items of SRS identified using forward feature selection. Duda et al. [53] validated the findings of Duda et al. [36] with crowdsourced data to improve the model’s capability on ‘real-world’ data, and the findings revealed that LDA outperformed LR and SVM by achieving an AUC of 0.89 with 15 items. Despite the high metrics reported by the studies, based on the standard clinical procedures for ASD diagnosis, the ML models are neither clinically sufficient nor readily implementable for real-life use.

Similarly, Wall et al. [35] compared the performance of 15 different ML algorithms on AGRE, SSC, and AC datasets and found ADTree to outperformed other models by utilizing 7 of the 93 items contained in the ADI-R in classifying ASD with 99.9% accuracy. In a similar study by Wall et al. [34], ADTree outperformed 17 comparative models by achieving 100% accuracy with 8 of the 29 items in Module 1 of ADOS. Moreover, Duda et al. [39] demonstrated the superior performance of ADTree in achieving 97% classification accuracy with a 72% reduction in ADOS-G items. Nonetheless, Levy et al. [50] and Kosmicki et al. [37] reduced the items of ADOS using sparsity/parsimony enforcing regularization and stepwise backward feature selection techniques, respectively, and reported the superior performance of LR and SVM over other ML algorithms. Specifically, in the study by Levy et al. [50], with at most 10 features from ADOS’s Module 3 and Module 2, AUC of 0.95 and 0.93 was achieved, respectively. While Kosmicki et al. [37] recorded an accuracy of 98.27% and 97.66% with 9 of the 28 items from module 2, and 12 of the 28 items from module 3, respectively. Recently, Küpper et al. [40] utilized ADOS data from a clinical sample of adolescents and adults with ASD and reported good performance of SVM on fewer items reduced using the recursive feature selection technique. The foregoing studies have demonstrated how ML-enable ASD screening and diagnostic models could be developed and evaluated. However, numerous challenges associated with the behavioral assessment instruments, data repositories, and applied data intelligence algorithms need to be understood and addressed.

Although ML-based approaches are data-centric and are expected to improve objectivity and automation [66], with the global rise in ASD cases, the capacity to quickly and accurately assess ASD requires a careful understanding of the conceptual basis of the assessment instruments, as well as their relevance to the logical concepts of the ML algorithms. Nonetheless, discrepancies within the data repositories, such as data imbalance, limit the clinical relevance of the high evaluation metrics reported in the studies [26,27]. For instance, Torres et al. [67] studied the statistical properties of ADOS scores from 1324 records and identified various factors that could undermine the scientific viability of the scores. Particularly, the empirical distributions in the generated scores break the theoretical conditions of normality and homogeneous variance, which are critical for independence between bias and sensitivity. Thus, Torres et al. [67] suggested readjusting the scientific use of ADOS, due to the variation in the distribution of the scores, lack of appropriate metrics for characterizing changes, and the impact of both on sensitivity-bias codependencies and longitudinal tracking of ASD. In essence, the applied data intelligence algorithms, and the resulting models, missed the human knowledge upon which the assessment instruments were built and applied by the professionals [25]. Additionally, most of the studies overlooked the inherent limitations associated with the dimensionality reduction techniques, and the assessment instruments [7,8,9]. Thus, the need for ascertaining the clinical relevance of the data-centric approaches and readjusting the scientific use of the assessment instruments remains. Obviously, in the future, it can be said that the trend in the application of ML in the behavioral assessment of ASD will go on. On the other hand, the pressing demands for cost-effective assessment of ASD remain. Thus, future studies need to revisit the relevance of the data collection instruments to ML algorithms.

## 5. Conclusions and Recommendations

Machine learning has been broadly applied in the behavioral assessment of ASD based on a variety of data types as input to data-intelligence algorithms. Commonly utilized inputs include the items of screening tools, such as ADI-R and ADOS-G. Popular ML algorithms used are SVMs, variants of the decision trees, random forests, and neural networks. However, the multitudes of challenges in accurate ASD assessments are yet to be addressed by the suggested machine learning approaches. Specifically, the high metrics achieved with the data-intelligence techniques have not guaranteed the clinical relevance of the ML models. Additionally, the commonly used evaluation measures of classification accuracy, specificity, and sensitivity, among others cannot sufficiently reflect the human knowledge applied by professionals in assessing behavioral symptoms of ASD. Consequently, understanding the clinical basis of the assessment tools and the logical concepts of the data-intelligence techniques will lead to promising studies on the real-life implementation of cost-effective ASD assessment systems. The novelty in the present review is that while previous literature reviews focused on the performance of various data intelligent techniques on different data sets, this work systematically reviewed the literature and provide a definitive explanation on the relevance of the reported findings toward the real-life implementation of the ML-based assessment systems. The authors hope that the findings of this systematic literature review will guide researchers, caregivers, and relevant stakeholders on the advances in ASD assessment with ML.

Nonetheless, a few of the limitations associated with the present work include overlooking other non-English documents. Thus, possible excellent studies reported in other languages might have been missed. Secondly, the search filters spanned ten years and were limited to the four scientific databases mentioned. Furthermore, the records retrieved relied on the few search terms utilized in the search query. Therefore, relaxing the search filters across additional databases could yield additional relevant studies. Lastly, the present review considered only full-text online journal articles. Consequently, the findings are limited to the studies included. The future research agenda will be based on relaxing the search criteria to incorporate other scholastic databases for further comparative results. In addition, future studies could relax the search filters to include books, conference papers, and so on. Noteworthy, to build on or replicate the reviewed studies, future research should explore data-intelligence techniques that will achieve not only excellent evaluation metrics, but also adhere to the conceptual basis upon which professionals diagnose ASD.

## Figures and Tables

**Table 1:** Inclusion and exclusion criteria of the study.

Inclusion Criteria
Journal articles published in the English language
Documents published within the last ten years from 2011 to date
Full-text papers that are accessible and downloadable
Studies that utilized behavioral data
Studies that employed machine learning as the main technique
Studies that considered autism as the main disorder assessed
Exclusion criteria
Papers that are written in other languages
Duplicated papers
Full-text of the document is not accessible on the internet
The study aim is not clearly defined
Studies that are not relevant to the stated research question
Relevant studies, but machine learning is not the main method
Relevant studies, but autism is not the main disorder assessed
Conferences papers, editorial materials, and literature reviews
Studies that utilized data from either brain imaging, genetic, or physical/metabolic biomarkers.
Intervention studies

## Data Availability

Data sharing is not applicable to this article.
